# Microembolism Induces Anhedonia but No Detectable Changes in White Matter Integrity in Aged Rats

**DOI:** 10.1371/journal.pone.0096624

**Published:** 2014-05-08

**Authors:** Christina L. Nemeth, David A. Gutman, Waqas Majeed, Shella D. Keilholz, Gretchen N. Neigh

**Affiliations:** 1 Department of Psychiatry and Behavioral Science, Emory University, Atlanta, Georgia, United States of America; 2 Department of Physiology, Emory University, Atlanta, Georgia, United States of America; 3 Department of Biomedical Informatics, Emory University, Atlanta, Georgia, United States of America; 4 Coulter Department of Biomedical Engineering, Emory University/Georgia Institute of Technology, Atlanta, Georgia, United States of America; 5 LUMS, School of Science and Engineering, Department of Electrical Engineering, Lahore, Pakistan; St Michael's Hospital, University of Toronto, Canada

## Abstract

Microvascular disease leads to alterations of cerebral vasculature including the formation of microembolic (ME) strokes. Though ME are associated with changes in mood and the severity and progression of cognitive decline, the effect of ME strokes on cerebral microstructure and its relationship to behavioral endpoints is unknown. Here, we used adult and aged male rats to test the hypotheses that ME lesions result in subtle changes to white and gray matter integrity as detected by high-throughput diffusion tensor imaging (DTI) and that these structural disruptions correspond to behavioral deficits. Two weeks post-surgery, aged animals showed depressive-like behaviors in the sucrose consumption test in the absence of altered cerebral diffusivity as assessed by ex-vivo DTI. Furthermore, DTI indices did not correlate with the degree of behavioral disruption in aged animals or in a subset of animals with observed tissue cavitation and subtle DTI alterations. Together, data suggest that behavioral deficits are not the result of damage to brain regions or white matter tracts, rather the activity of other systems may underlie functional disruption and recovery.

## Introduction

Microvascular pathology is common in the aged population and the incidence of microvascular disease is growing [1]. Small vessel pathology, including within the context of microvascular pathology, and microvascular events (e.g. thickening of arterial walls, microvascular lesions, and microembolic strokes [2], [3]) are known contributors to depressive behaviors late in life [4]–[10], and have been implicated in the progression of cognitive impairment and Alzheimer's disease [11], [12]. Despite these links and the high correlation between microvascular disruption and behavioral deficits, the mechanisms behind this relationship remain a topic of debate [13]. One study found that an estimated 94% of patients with late onset depression (first episode after age 65) exhibited diffuse cerebral lesions [14]. Similar findings linking the presence of infarcts to behavior [7], [13], [15] led to the development of the Vascular Depression Hypothesis which posits that changes to cerebral vasculature precipitate behavioral changes, such as depression and dementia [6], [14]. Due to the slow progression of these symptoms, the phenotypically silent nature of these lesions, and the difficulty of directly addressing this relationship in the clinic, establishing a cause and effect relationship has been difficult. Finally, because ME lesions are typically detected following a larger ischemic event or after death, preventative measures and treatment strategies are greatly hindered.

Rodent microembolism (ME) models have been used to successfully recapitulate the cognitive deficits of microvascular pathology [16], [17] as well as the clinical features of stroke [18], [19]. Importantly, recent work has shown that these lesions are sufficient to induce anhedonic- and anxiety-like behaviors, as well as impairments in spatial memory in adult male rats [20]. In these studies, however, the relationship between microsphere lesions and behavioral disruption were not paralleled by cellular death, macrophage activation, or astrocyte activity measured by conventional histology. Therefore, the current study employed diffusion tensor imaging (DTI) as a method of assessing microstructural differences in tissue to complement previous histological findings in this model. In our study, we adopted high-throughput *ex vivo* imaging, a novel method to more efficiently image multiple perfused brains simultaneously [21], [22]. Assessment of DTI indices in microsphere lesioned brains may provide a more translatable correlate compared to slice histology.

DTI is a powerful tool that allows for the assessment of microstructural organization, orientation of white matter tracts, and provides detailed information on the integrity of biological tissues. DTI depends on the anisotropy and diffusivity of water molecules through tissue fibers, and thus metrics of DTI, fractional anisotropy (FA) and mean diffusivity (MD), can be used as indices of healthy tracts and tissue microstructure. The sensitivity of DTI allows for the detection of subtle changes in cell, axon, and myelin morphology and has become a useful tool in predicting functional outcome following stroke and other brain injuries [23], [24]. Despite growing popularity of DTI for neuroimaging, little is known about the relationship between DTI-derived measures and affective behavior in the context of microvascular pathology. Further, to the best of our knowledge, this has not been examined in an aged model.

We have previously established that the induction of microsphere lesions results in behavioral disruption in adult animals that does not correlate with cell death, or macrophage/astrocyte activity as detected by conventional histology. Thus we sought to more closely explore the interactions of microvascular structure and normal aging. To accomplish this, we elected to compare the effects of ME infarction in adult and aged rats. Through the examination of behavior in aged rodents, we tested the hypothesis that subtle ME-induced lesions result in structural changes to white matter integrity and gray matter tissue, as measured by DTI, that correspond to the manifestation of behavioral deficits.

## Materials and Methods

### Ethics Statement

All experiments were performed in accordance with the Institutional Animal Care and Use Committee (IACUC) of Emory University, the National Institutes of Health *Guide for the Care and Use of Laboratory Animals*, and *The Code of Ethics of the World Medical Association for experiments involving laboratory animals*. The protocol was approved by the IACUC of Emory University and all efforts were made to alleviate animal suffering.

### Animals

Adult (3 months of age) and aged (16 months of age, Charles River) male Wistar rats were pair housed until surgery. Rats were maintained on a reverse 14∶10 light:dark cycle in a temperature and humidity controlled AAALAC-approved facility. To promote healthy aging prior to the start of the study, aged rats were provided cage enrichments and maintained at a healthy weight by limited food ration. At the time of ME or SHAM surgery (described later), and throughout the duration of the study, cage enrichments were removed and food and water were available *ad libitum* regardless of age.

### Surgery

Following a minimum one week acclimation period, rats were randomly assigned to SHAM (adult n = 7; aged n = 7) or ME (adult n = 9; aged n = 9) surgical groups. Animals were anesthetized with isoflurane and a neck incision was made to locate the common, internal, and external carotid arteries. The common carotid artery was isolated and ligated with suture and the external carotid artery was ligated at the bifurcation with the internal carotid artery. Microspheres (New England Nuclear Inc., Boston, MA; 50 µm in diameter; suspended in 10% Dextran and 0.01% Tween in isotonic saline; PerkinElmer Instruments; Shelton, CT; ≈2500 spheres in 50 µL) were injected using a 30 G needle to the left internal carotid artery. Pressure was applied to the injection site until bleeding stopped, at which point ligations were released and blood flow was allowed to return to normal. SHAM animals underwent an identical preparation and ligation procedure without an injection. The wound was closed with surgical staples and antiseptic was applied. Surgical staples were removed on post-surgical day 5. Animals recovered in their home cage for fourteen days prior to assessment of depressive- and anxiety-like behaviors (aged animals) and imaging (adult and aged animals).

### Behavioral Testing

In the current experiment, aged animals were tested beginning at fourteen days post-ME in the open field paradigm and the sucrose consumption test to determine the effect of ME infarction on susceptibility of aged animals to behavioral deficits. Behaviors were assessed as previously described [20] and began on Day 14, with one test conducted per day.

In order to assess anxiety-like behavior, the open field test [25] was conducted during the animals' dark phase, and was videotaped and hand scored by an observer blind to surgery groups. Time spent in the center (60 cm×60 cm of 1 m^3^ open box) versus the periphery of the box, or central tendency, was interpreted as exploratory anxiety [25] and locomotor activity was assessed by counting the total number of squares crossed. The open field test lasted ten minutes and started with placement of the rat in the center of the apparatus.

The sucrose consumption test measures animals' preference for a sucrose solution over tap water and serves as an indicator of reward state, such that decreased sucrose consumption reflects an anhedonic state [26]. Sucrose consumption was assessed over 48 hours (Days 15–17 post-ME) in the animals' home cages. During this time, rats had equal access to a 0.8% sucrose solution and tap water. A 24-hour habituation period was followed by a 24-hour evaluation period. Bottle presentation was reversed after the first 24 hours to prevent side bias [20].

### Tissue Preparation

In order to reduce the time required to scan the brains, we adopted a high-throughput imaging paradigm used by Gutman *et al.* (2012). Following behavioral testing (post-ME day 18), all animals were administered Euthasol and transcardially perfused with 4% paraformaldehyde. Brains were then removed and stored at 4°C until they were embedded in an agarose matrix for imaging. Three to four rat brains were embedded into each of seven tubes for imaging. The embedding mixture was composed of a matrix of 1% agarose (Sigma, St. Louis, MO) doped with an insoluble mixture of 1 mM gadolinium oxide (Fisher Scientific, Pittsburgh, PA); the gadolinium serves to suppress the signal from the agarose, providing better separation of the brains from the background.

### Imaging

All imaging experiments were performed on a Bruker 9.4T horizontal scanner using a 72 mm volume coil (Bruker, Billerica, MA). Adult and aged SHAM and ME rat brains were randomly assigned to imaging tubes. For each tube of brains, T2-weighted images were first acquired at 161 micron isotropic resolution (echo time [TE]  = 25 ms, matrix 256×512, 12 averages, ∼16 hours scan time). DTI images were acquired using a spin-echo based sequence with 200 micron isotropic resolution (TE = 26.9 ms, TR = 27.5 s, matrix size of 256×128, ∼55–60 axial slices, 64 gradient directions with b  = 2000 s/mm^2^, 3 images with b  = 0, scan time  = ∼61 hours).

### Segmentation and Registration

**Figure 1:**
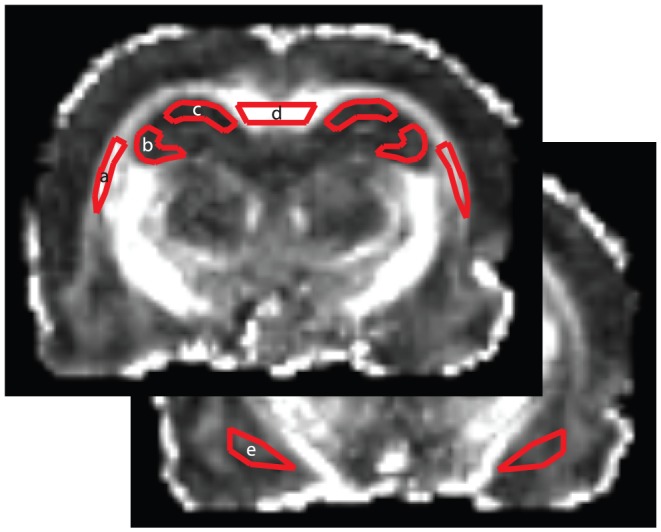
Volume of interest (VOI) regions were individually defined on an image stack to create a 3D image on which analysis was conducted. The following regions were measured for fractional anisotropy (FA) and mean diffusivity (MD): external capsule (a), CA3 subfield of the hippocampus (b), CA1 subfield of the hippocampus (c), genu of the corpus callosum (d), and the amygdala (e). For all regions, except the corpus callosum, the corresponding contralateral regions were also measured.

Prior to further processing, the images from each scan that contained one or more individual rat brains were manually segmented into individual files. For both the T2-weighted and DTI-scans, masks were generated for each individual rat brain using BET, (FSL, www.fmrib.ox.ac.uk/fsl) followed by manual editing to generate individual brain masks for each animal. A study-specific high-resolution rat template was generated by nonlinearly registering each T2-weighted brain to a single reference image, and then creating a composite image (FNIRT,www.fmrib.ox.ac.uk/fsl). A transformation matrix for each rat DTI dataset to this rat-standard space was then generated using FLIRT (FSL, www.fmrib.ox.ac.uk/fsl) using a 12-DOF affine warp. Brain region and white matter tract volumes of interest (VOIs) areas were subsequently defined on each individual reference image (9 total VOIs including: the genu of the corpus callosum, and the ipsilateral and contralateral regions of the amygdala, external capsule, CA1 and CA3 subfields of the hippocampus; Figure 1). The mean intensity from the FA map was then extracted from each VOI and averaged as described below. All image processing was conducted by an investigator blinded to all treatment groups.

### Data Analysis

All data were assessed for normality and equal variance prior to statistical analysis. Animal weights were assessed using a three-way repeated-measures ANOVA with age (adult vs aged), surgery (SHAM vs ME), and days post-surgery (1 vs 7 vs 18) as factors. For each age group, further group differences were delineated using a two-way repeated-measures ANOVA with surgery (SHAM vs ME) and day (1 vs 7 vs 18) as factors. Sucrose consumption was assessed using a two-way repeated-measures ANOVA with surgery (SHAM vs ME) and day (Day 1 vs Day 2) as factors. Open field behavior was averaged by group (SHAM vs ME) and compared using a student's t-test.

For each VOI, FA and MD values were computed and averaged by group. For both adult and aged animals, values measured for each region and tract were analyzed by a two-way repeated-measures ANOVA with surgery (SHAM vs ME) and hemisphere (ipsilateral vs contralateral) as factors. FA and MD values of the genu of the corpus callosum were compared to SHAM treated animals using a student's t-test. Differences in lesion frequency were determined using a two-sample t-test of proportions. For all comparisons, results were considered significant when *P*<0.05. All results are reported as means ± the standard error of mean (S.E.M.) and all analyses were performed with GraphPad Prism 5.

## Results

### Microsphere injection induces depressive-like behaviors in aged rats with no effect on overall health

**Figure 2:**
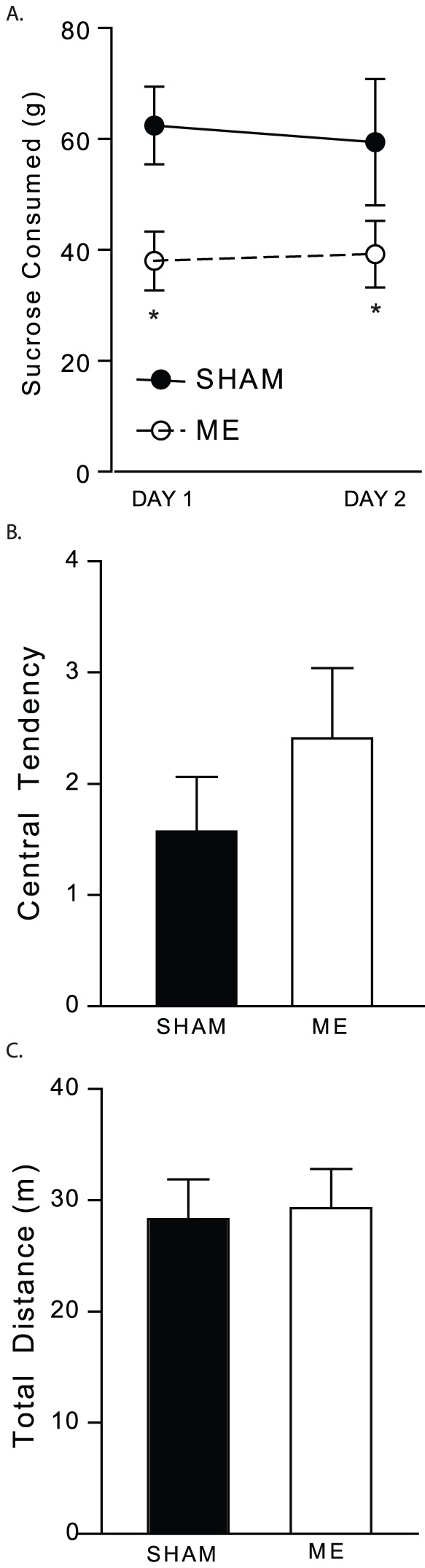
Aged SHAM and ME animals were evaluated in the sucrose consumption test and open field for depressive- and anxiety-like behaviors, respectively. (A) Aged ME animals in the sucrose consumption test consumed less sucrose over a two day period compared to aged SHAM animals (* indicates *P*<0.05). (B,C) In the open field, no differences existed in the time spent in the center of the arena, or in the total distance traveled between aged SHAM and ME animals. SHAM n = 7; ME n = 9. Error bars indicate standard error of mean (S.E.M.).

Sucrose consumption was measured as an index of anhedonic-like behaviors in aged animals over a two day period in the home cage. A two-way repeated measures ANOVA demonstrated a main effect of surgery, such that aged ME rats consumed less sucrose over a 48 hour period compared to aged SHAM animals (Figure 2A; F(1,14)  = 4.966, *P*<0.05). This difference in consumption is not due to differences in body weight between the groups, for weight in each age group did not differ as a function of surgery (post behavior weights: adult SHAM 501.0 ± 14.81; adult ME 515.56 ± 12.10; aged SHAM 668.71 ± 24.87; aged ME 671.33 ± 20.32). Although aged animals weighed significantly more than adult animals (*P*<0.05), a three-way repeated measures ANOVA revealed no interaction between age, surgery, or post-surgery day (F(2,84)  = 0.095, *P*>0.05). Furthermore, a two-way repeated measures ANOVA of adult (F(1,14)  = 0.417, *P*>0.05) or aged (F(1,14)  = 0.016, *P*>0.05) animals revealed no effect of surgery.

Additionally, analysis of behavior in the open field showed no differences in the amount of time spent in the center of the arena between aged SHAM and ME animals (Figure 2B; *P*>0.05). Similarly, no differences in total mobility in the open field were found between surgery groups, suggesting no physical impairment (Figure 2C; *P*>0.05).

### Microsphere injection does not cause long term alterations in tract or tissue diffusivity

**Figure 3:**
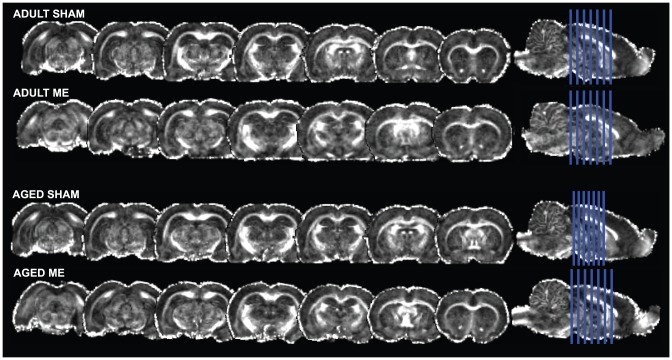
Aged and adult SHAM and ME perfused brains were secured in agarose and simultaneously scanned by DTI using a Bruker 9.4T horizontal scanner. Figure shows representative multislice display of SHAM, adult ME, and aged ME brains.

**Figure 4:**
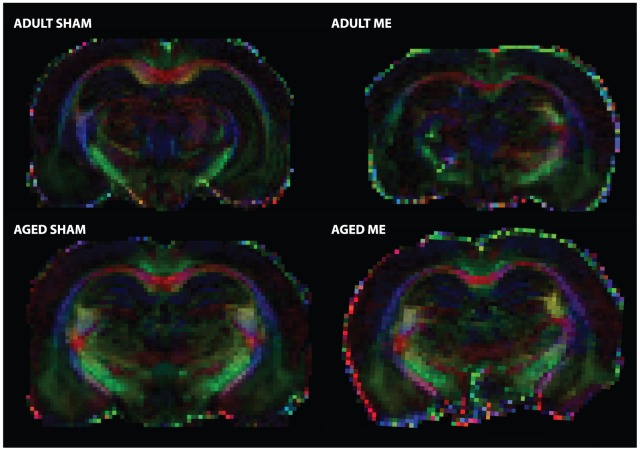
Representative color maps of fractional anisotropy (FA) values for adult- (top) and aged- (bottom) SHAM and ME rats. Colors represent tract strength and directionality; red, green, and blue, indicate transverse, rostrocaudal, and anterior-posterior, respectively.

FA and MD serve as indices of pathology in diffusion tensor scanned brains. Figure 3 shows representative high resolution images of adult SHAM, adult ME, and aged ME rat brains (for all brains, please see Figure S1). At more than two weeks following surgery, aged rats exhibited no differences in FA in either the CA1 or CA3 subfields of the hippocampus or in the amygdala compared to aged SHAM animals. Analysis of white matter tracts revealed similar findings; no differences were detected in the strength of directionality (FA) in the external capsule as compared to the contralateral hemisphere or in the corpus callosum as compared to adult or aged SHAM animals (Figure 4 shows representative FA map; adult and aged group means presented in Table 1; *P*>0.05).

**Table 1:** Fractional anisotropy (FA; top) and mean diffusivity (MD) values of adult and aged rats by region.

	ADULT				AGED			
FA	SHAM	±SEM	ME	±SEM	SHAM	±SEM	ME	±SEM
Ipsi- CA1	0.1198	0.001	0.1029	0.010	0.1429	0.018	0.1176	0.070
Contra- CA1	0.1014	0.004	0.1168	0.005	0.1500	0.016	0.1186	0.007
Ipsi- CA3	0.1231	0.023	0.1816	0.063	0.1226	0.005	0.1214	0.006
Contra- CA3	0.1150	0.017	0.1297	0.005	0.1235	0.005	0.1234	0.006
Ipsi- Amygdala	0.1559	0.001	0.1389	0.016	0.1331	0.012	0.1458	0.093
Contra- Amygdala	0.1268	0.007	0.1375	0.011	0.1322	0.010	0.1485	0.088
Ipsi- Ext. Capsule	0.4011	0.019	0.3603	0.015	0.3929	0.019	0.3868	0.015
Contra- Ext. Capsule	0.3756	0.037	0.3035	0.034	0.3872	0.017	0.3920	0.017
Corpus Callosum	0.5255	0.044	0.4951	0.032	0.4966	0.025	0.5308	0.024

	ADULT				AGED			
MD	SHAM	±SEM	ME	±SEM	SHAM	±SEM	ME	±SEM
Ipsi- CA1	0.7150	0.014	0.6205	0.075	0.5912	0.038	0.6356	0.068
Contra- CA1	0.7535	0.001	0.6810	0.031	0.5456	0.054	0.6433	0.076
Ipsi- CA3	0.5947	0.081	0.4950	0.012	0.5560	0.040	0.5678	0.050
Contra- CA3	0.5857	0.088	0.7290	0.122	0.5428	0.033	0.5452	0.048
Ipsi- Amygdala	0.6930	0.014	0.6478	0.032	0.5623	0.061	0.5707	0.063
Contra- Amygdala	0.6850	0.003	0.7135	0.010	0.5585	0.064	0.6097	0.079
Ipsi- Ext. Capsule	0.4253	0.056	0.4480	0.018	0.4177	0.018	0.4128	0.049
Contra- Ext. Capsule	0.4653	0.080	0.4280	0.019	0.4353	0.015	0.3992	0.039
Corpus Callosum	0.4753	0.041	0.4812	0.120	0.4052	0.029	0.4814	0.073

Table shows values of fractional anisotropy (FA; top) and mean diffusivity (MD, ×10^-3^ (mm^2^/s); bottom) of SHAM and microembolism (ME) animals for adult (left column) and aged (right column) animals. For each age group, no significant differences in FA or MD were detected across the nine brain regions analyzed.

As expected, analysis of MD paralleled FA data. Gray matter diffusivity was unchanged across hemispheres in the CA1, CA3, and amygdala, with no effect of age. MD values for the external capsule and corpus callosum were unchanged by surgery, regardless of age (Table 1; *P*>0.05).

**Figure 5:**
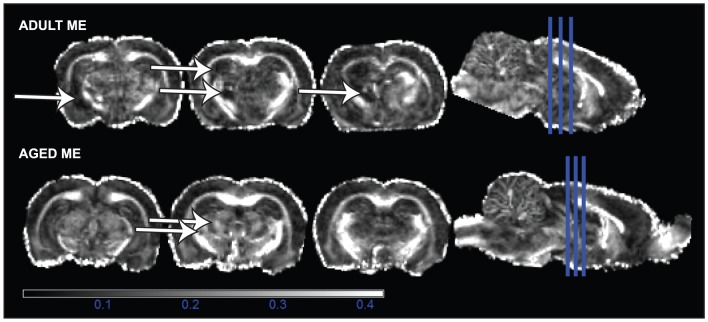
Representative coronal brain sections from adult (top) and aged (bottom) ME rats show cavitation in areas of the ipsilateral amygdala, hippocampus and thalamus. Scale bar represents values of FA.

Visual observations of tissue cavitation were noted in a subset of ME animals comprising 3 adult and 2 aged rats (Figure 5). Table 2 outlines lesion location and frequency within adult and aged animals. A two-sample t-test between proportions revealed no differences in the lesion frequency between adult and aged rats in any brain region observed (P>0.05). Analysis of animals with tissue cavitation demonstrated reductions in FA in the external capsule of adult ME animals compared to age-matched SHAM operated controls (main effect of surgery: F(1,8)  = 5.651, *P*<0.05). No changes in FA or MD were observed in any region of aged ME animals as compared to aged-matched SHAM operated controls (data not shown, *P*>0.05). Furthermore, comparisons of the cavitated subset of aged animals to either non-damaged aged ME animals or aged SHAM animals was not predictive of behavior (data not shown, *P*>0.05).

**Table 2:** Summary of lesion location and infarct rate.

	% Adult	% Aged	% Total
Lesions			
Amygdala	14.3	33.3	23.1
Caudate	29.6	50.0	38.5
Cortex	29.6	50.0	38.5
Hippocampus	14.3	33.3	23.1

Lesion location and frequency are listed for adult ME and aged ME animals as visible by T2-weighted scans. No patterns were evident in the frequency or location of lesions, values listed are not interdependent.

## Discussion

Our results demonstrate that the induction of diffuse microsphere lesions is not sufficient to produce lasting alterations to metrics of DTI in adult or aged animals eighteen days following the procedure. However, consistent with previous findings in adult rodents [20], we found that aged animals exhibit increased depressive-like behavior as a result of ME damage. These data indicate that ME lesions do not affect the pathology of cerebral tracts or tissue and that damage does not predict behavioral disruption.

High-throughput e*x vivo* DTI analysis of ME-treated aged rodent brains revealed no changes in tract strength or gray matter tissue integrity (as measured using FA and MD) in any brain region measured. Selection of VOIs for the current experiment was based on previous observations of region-specific tissue susceptibility related to ME infarction [20], stroke [27], and cerebral hyperintensities in the presence of small vessel disease [28]. Following acute strokes in humans and experimental stroke in rodents, it is reported that in these regions of susceptibility, ischemic lesions, and cellular swelling peak by seven days and normalize within weeks [24], [29]. Similarly, indices of DTI, such as FA and MD, change parallel to the evolution of damage following ischemic injury. In the current study, we can speculate that cellular swelling and edema have subsided prior to imaging, which may have led to normalization of DTI measures. Furthermore, we do not suspect that paraformaldehyde fixation or tissue preparation related to *ex vivo* imaging resulted in tissue compromise or confounded results, as previous studies have reported microstructural stability and high quality data sets following fixation and postmortem imaging [21], [22]. In fact, *ex vivo* imaging offers the ability to obtain high resolution data, with high signal to noise ratio, without motion artifacts over long scan times- resolution that is seldom obtained from waking animals [21], [30].

Though we might expect that the susceptibility of the aged human brain to ME lesions would result in a greater degree of damage to aged animals in the current study [1], [31], [32], this finding has been poorly replicated in rodent models and differences in infarct size between young and aged rodents are rarely observed [33]. Aged rats had a higher proportion of cavitation compared with adult rats; however, this difference failed to reach significance and did not correlate to any other measured metric. Further, in one examination of adult and aged rats following microsphere injection, adult rats showed neurological deficits shortly after the procedure, while aged animals' deficits were gradual and far less severe [34]. Though the behavioral manifestation of stroke-related impairment in rodents appears to contrast clinical data, the neurochemical factors influencing poor stroke recovery among the elderly human population are paralleled in rodents and are characterized by increased rates of cell death and scar formation following trauma, in addition to slower response rates of neurotrophic and neuroprotective mechanisms [35]. Despite discrepancies in the rates of actions between the adult and aged brains, in long-term assessments (greater than 1 week) of recovery, responses to ischemic events appear to conclude at the same time [35] and our findings likely reflect the stabilization of damaged areas [36].

Diffusivity in the adult rodent brain was consistent with aged findings, such that group differences in FA and MD did not differ from aged-matched SHAM operated controls. Similarly in this cohort, we believe that the normalization of values reflects recovery of acute edema and cellular responses to ME procedures. In the adult population, we observed variable instances of vasogenic edema that develops into cavitation [20], [37]. In fact, the ratio of cavitated to undamaged brains in our experiment is consistent with previous reports of microsphere injection models in our lab [20] and others [36], [37]. In this cohort of cavitated animals, adult, but not aged animals, exhibited reductions in FA in the external capsule only, supporting previous work that suggests the external capsule to be more sensitive to ischemic damage [27], therefore quantifiable damage to the external capsule is evident in more severe instances of ME infarction. Reductions of FA in the external capsule indicate cell membrane and myelin disruption, and demonstrate our ability to detect changes in DTI metrics following microsphere damage in adult and aged rats.

Aged rodents in the current experiment exhibited increased depressive-like behaviors more than two weeks following ME procedures. We elected to use aged animals in this assessment because aged rodent models of microvascular pathology are clinically more relevant than models involving young adult animals [35] and this would allow us to extend our previous findings that ME alters affective-like behaviors in adult male rats [20]. Though these data support the Vascular Depression Hypothesis [6], a full characterization of behavioral deficits was not possible in aged animals during the current experiment as a lack of movement in the aged cohort confounds interpretation of these and other behavioral results that rely on motor activity. In all, adult and aged animals manifest similar behavioral disruptions following ME procedures in the absence of altered metrics of DTI, suggesting that adult animals may be a sufficient population in which to study microvascular events without the confound of reduced motor activity.

Using DTI, we have determined that ME infarcts are not sufficient to alter FA and MD in a manner that is associated with the manifestation of behavioral disruption. As stated, in the subset of animals with observed cavitation, we were able to detect minimal structural damage with DTI demonstrating that our ME procedure induces long lasting structural damage visible on the macro scale in a subset of the animals. Despite the presence of cavitation in a proportion of animals, behavioral assessment of these cavitated animals compared to SHAM or non-damaged ME animals did not differ, suggesting that behavioral changes were not mediated by overt damage as assessed by DTI, findings consistent with previous histological comparisons [20], [38]. Given that previous work by our group [20], and others [16], [39], [40], has demonstrated that traditional histological analyses of microsphere-induced damage are inconclusive and may not be sensitive enough to detect microsphere-induced damage, we limited the current hypothesis to include only metrics of DTI. Specifically, we hypothesized that the gestalt assessments of brain morphology that are possible with DTI would be able to detect microsphere-induced structural changes in the brain. Based on our findings, we conclude our methods were appropriate for the detection of FA or MD changes using DTI. In fact, our data may reflect tissue stabilization more than two weeks following injury. Though independently, values of FA can reflect white matter changes, FA is determined from a ratio of eigenvalues and should be used in conjunction with additional parameters (mean diffusivity, radial and axial diffusivity) [24] to provide a more thorough understanding of tissue structure following damage. MD is affected by myelin disruption, tissue cavitation, edema, and cell death, all factors that evolve temporally, and complement information provided by FA in diffusion based studies [24], [41]. Furthermore, while other DTI indices are available, the use and interpretation of radial and axial diffusivity is somewhat controversial and not well understood [27], [42] and were deemed unnecessary for the current analyses. MD and FA remain the most frequently reported indices of tissue and tract integrity, and FA appears most predictive of stroke outcome [23], thus making it appropriate for our analyses.

The present experiments tested the hypotheses that 1) adult and aged animals experience ME-induced structural damage as measured by DTI; and 2) that these structural damages correlate to the manifestation of behavioral disruption in aged rats. While aged animals showed depressive-like behaviors following ME, as compared to SHAM operated animals, this was not paralleled by altered DTI metrics in the ME aged group. In adult brains and aged brains, no group level differences were observed in FA or MD following ME procedures, suggesting ME infarction to be too acute to generate permanent structural alterations to tract or tissue integrity, as detectable by DTI. In conclusion, our results indicate that the subtle presentation of ME lesions does not alter metrics of DTI in the rodent brain, and thus factors that influence FA and MD, as measured by DTI, do not contribute to the manifestation of behavioral deficits following ME lesions. These findings are of importance because it suggests that the functioning of remaining cells and increased activity of other systems underlie behavioral perturbations following ME lesions and that the activity of these systems may be a better target for functional recovery following microvascular events. Future experiments will explore alternative hypotheses between microsphere damage and behavior.

## Supporting Information

Figure S1 **Complete scanning data are provided for adult SHAM, adult ME, aged SHAM, and aged ME rats included within the analyses of this study.**
(PDF)
