# Supplementary figures and images for: Microembolism Induces Anhedonia but No Detectable Changes in White Matter Integrity in Aged Rats
Source: PLoS One. 2014 May 8;9(5):e96624. doi: 10.1371/journal.pone.0096624 (PMC4014537; doi:10.1371/journal.pone.0096624)

ADULT SHAM

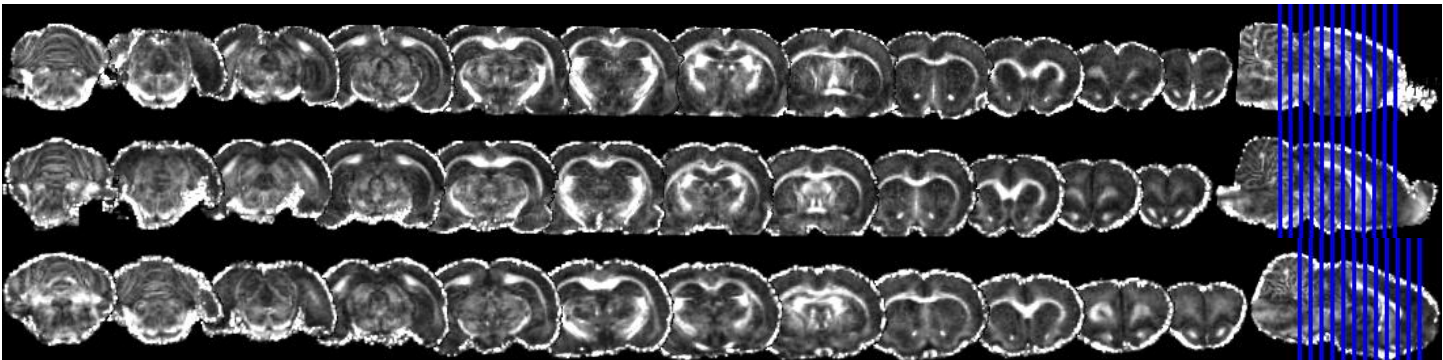

ADULT ME

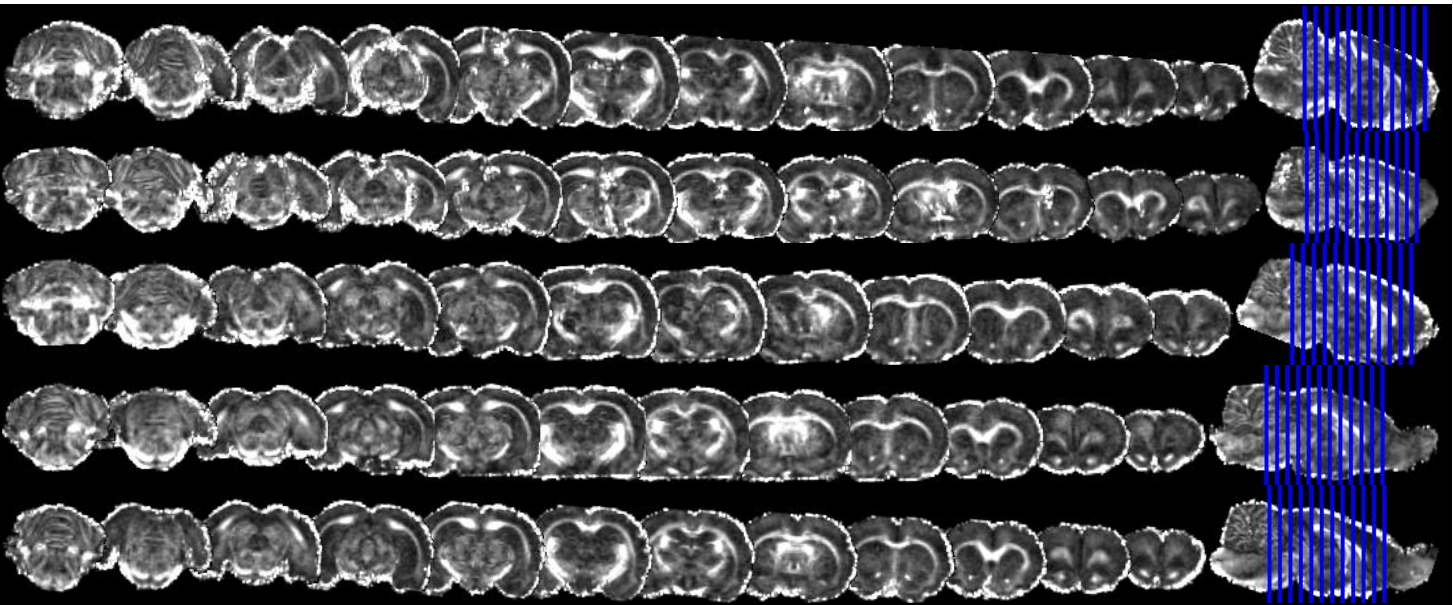

AGED SHAM

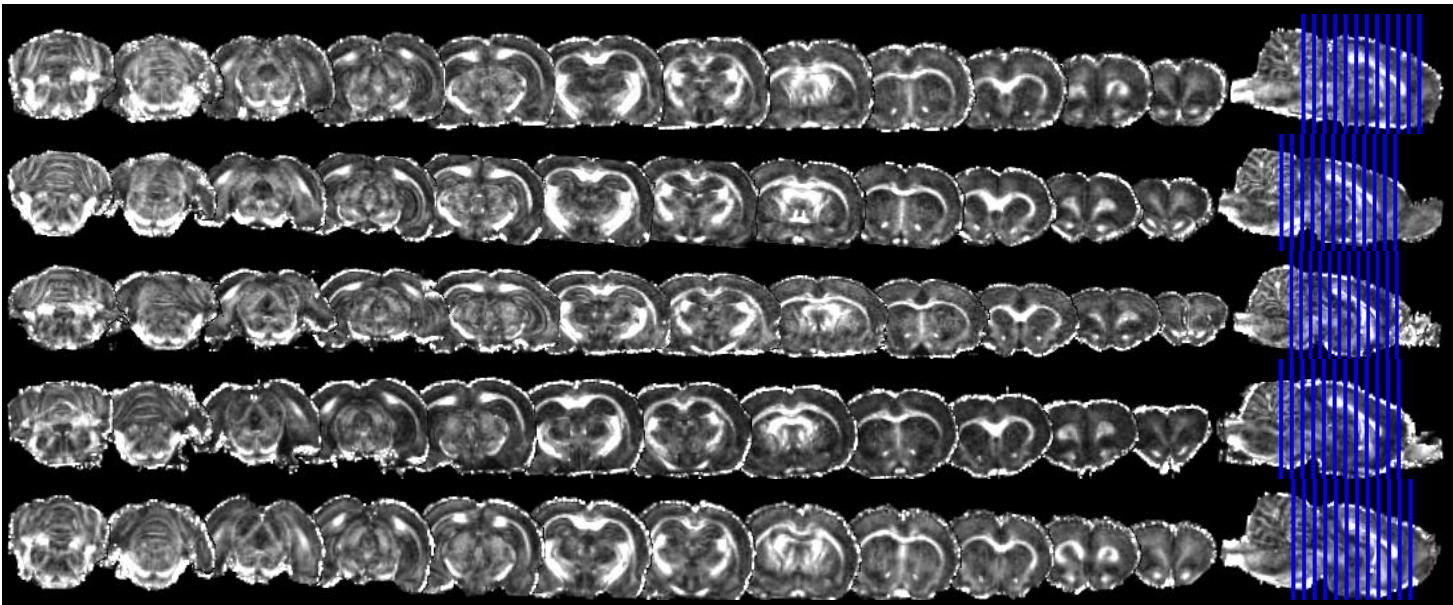

AGED ME

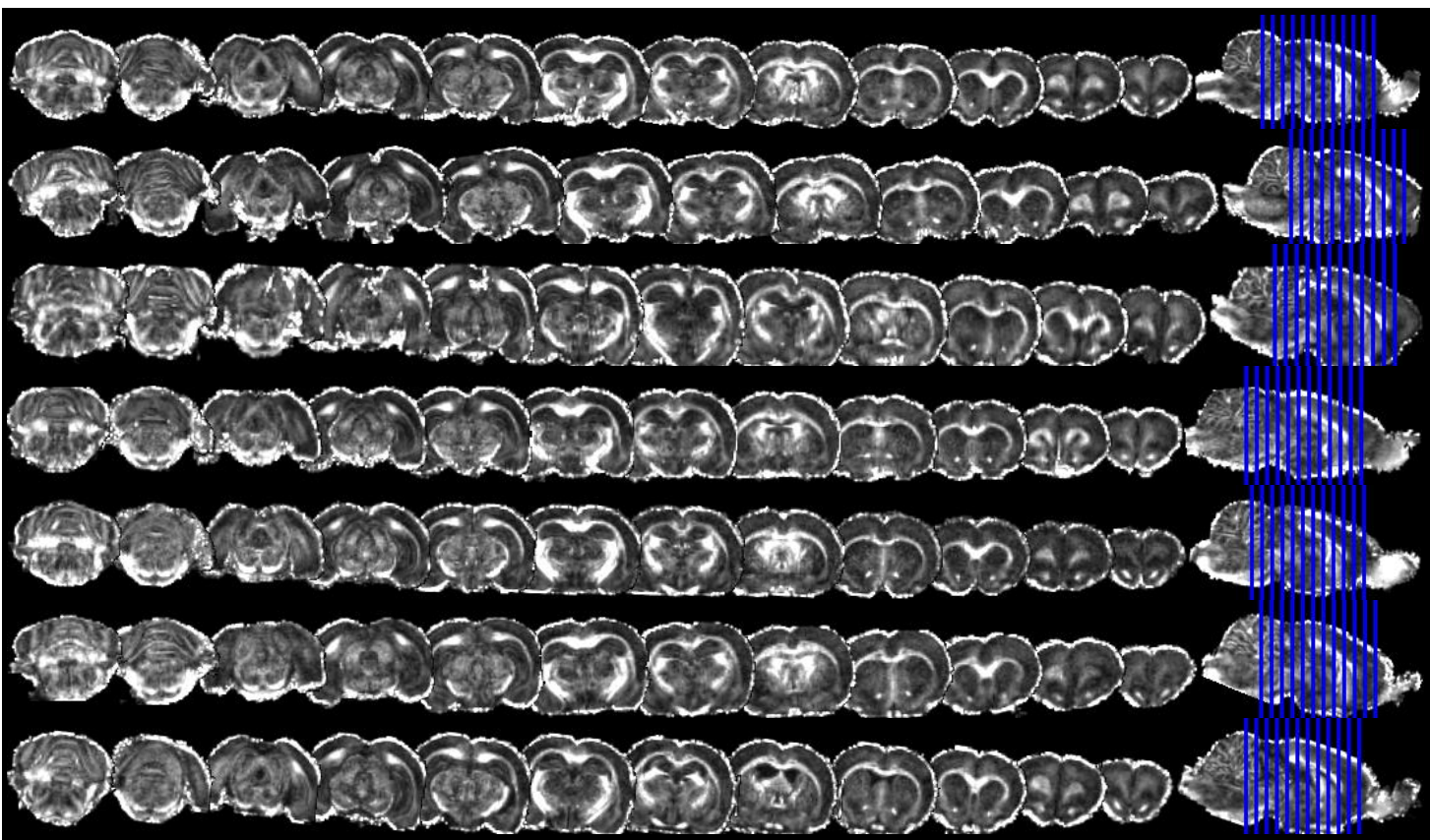

Supplement: Figure S1 — Complete scanning data are provided for adult SHAM, adult ME, aged SHAM, and aged ME rats included within the analyses of this study. (PDF) [file pone.0096624.s001.pdf]
